# The TDG protein environment connects active DNA demethylation with chromatin and RNA biology

**DOI:** 10.1007/s00018-025-05943-y

**Published:** 2025-11-25

**Authors:** Federica Richina, Faiza Noreen, Christina Bauer, Alain Weber, Christophe Kunz, Katarzyna Buczak, Simon D. Schwarz, Fabian Wu, David Schürmann, Primo Schär

**Affiliations:** 1 Department of Biomedicine, University of Basel, Mattenstrasse 28, Basel, 4058 Switzerland; 2 Swiss Institute of Bioinformatics, Basel, 4031 Switzerland; 3 Proteomics Facility Biozentrum, University of Basel, Spitalstrasse 41, Basel, 4056 Switzerland

**Keywords:** Base excision repair, DNA methylation, Epigenetics, Proteomics, BioID2-MS, RNA, Chromatin

## Abstract

Understanding the interplay between DNA methylation and gene expression remains a challenge. This study explores the proteome of active DNA demethylation in murine embryonic stem cells (mESC), focusing on the base-excision-repair (BER) step initiated by the Thymine DNA Glycosylase (TDG). Using BioID2 proximity labeling, we identified a TDG interactome encompassing four functional aspects: chromatin organization and transcription, chromosomal organization, RNA processing, and ribosomal biogenesis. We show specifically that TDG participates in a genome regulatory network involving chromatin remodelers and modifiers such as RUVBL2 and the H3K4 methyltransferase complex tethering factor HCFC1, consistent with the dysregulation of histone modifications observed in TDG-deficient cells. We also identified the paraspeckle components PSPC1 and NONO as TDG interactors, implicating TDG in RNA-mediated nuclear processes. This led us to show that TDG is an RNA-binding protein, interacting with long-noncoding RNAs (lncRNA), including the paraspeckle organizing lncRNA *Neat1*, previously reported to target TET proteins to genomic sites and to engage in R-loop regulation. We then demonstrate TDG’s ability to excise oxidized 5-methylcytosine in RNA:DNA hybrids, suggesting a role of active DNA demethylation in the regulation of R-loops. Our findings thus unveil a direct crosstalk between active DNA demethylation, chromatin modification and remodeling as well as RNA-genome interactions in mESC, providing avenues for future mechanistic investigations.

**Supplementary Information:**

The online version contains supplementary material available at 10.1007/s00018-025-05943-y.

## Introduction

Dynamic DNA methylation is widespread among multicellular organisms, constituting an essential mechanism to control gene activity, facilitating the differentiation into and maintenance of stable cell types and ensuring proper embryonic development [1]. DNA methylation is set and maintained by de novo and maintenance DNA methyltransferases (DNMT3A, DNMT3B, DNMT1) [2, 3], whereas DNA demethylation can be achieved by passive or active mechanisms that act either globally or in a locus-specific manner [4]. DNA replication-independent active DNA demethylation occurs at specific gene regulatory regions and is mediated by the action of TET dioxygenases and TDG-dependent DNA base excision repair (BER) [5–10]. The two-tiered process starts with the sequential oxidation of 5-methylcytosine (5mC) to 5-hydroxymethylcytosine (5hmC), 5-formylcytosine (5fC), and 5-carboxylcytosine (5caC) by TET proteins. 5mC oxidation is then followed by the excision of the 5fC and 5caC bases by the DNA glycosylase TDG, initiating a BER process that restores the unmethylated cytosine [10]. While this DNA methylation/demethylation cycle, e.g., dynamic DNA methylation, is well characterized mechanistically, its functional impact on chromatin and gene regulation has remained largely elusive. TDG (and TETs) deficiency in mice causes embryonic lethality, associated with a failure to properly establish cell type-specific gene expression during cell differentiation [5, 6, 11]. This deficiency in gene programming is paralleled by the occurrence of aberrant histone modification and, less pronounced, DNA methylation [5, 12], suggesting functional links between active DNA demethylation and chromatin landscaping in differentiating cells. To explore at the molecular level how the TET-TDG-driven active DNA demethylation machinery interacts and cooperates with chromatin and gene regulatory factors in chromatin landscaping, we investigated the protein proximity space of TDG.

Proteins interacting with TDG have been reported previously. These include factors of the DNA methylation machinery, such as DNMT3B [13], TET1 [10] and GADD45a [14], as well as SUMO proteins that modify TDG and regulate its DNA binding affinity [15–18]. Interactions of TDG with chromatin modifiers and transcriptional regulators have also been described and include histone acetyltransferase CREBBP/P300 [19, 20], p53 [21, 22], as well as retinoic acid, thyroid and estrogen receptors [23–26]. Notably, most of these interactions were discovered in either targeted, hypothesis-driven approaches (e.g., pull-down assays) or in genetic screens. Apart from yeast-two-hybrid approaches, co-purification attempts with TDG as a bait were less fruitful, presumably due to its biochemical and biophysical properties. Within cell nuclei, TDG resides both in the soluble and insoluble chromatin-associated fractions. While the soluble TDG fraction is easily accessible to analyse for protein interactions, the chromatin-bound and presumably active TDG fraction presents a challenge because its extraction requires high salt concentrations, detergents, and extensive sonication. These harsh conditions disrupt protein–protein interactions of interest and thus perturb conventional TDG-directed approaches for affinity-based co-purification [27].

To overcome this limitation, we took advantage of the BioID2-MS method [28, 29]. This method relies on the expression of TDG tagged with a promiscuous biotin ligase (BirA*), which can covalently biotinylate proteins in direct proximity (labeling radius ~ 10 nm). This approach also provides the advantage of capturing the interactome over a defined timespan, thereby reducing the risk of missing transient interactions associated with highly dynamic processes. Moreover, the direct biotin-labeling of proximity partners facilitates simple purification of interactors under denaturing conditions by streptavidin binding and, thus, does not require inefficient and selective protein extraction and retrieval procedures.

Using the BioID2-MS method, we identified the TDG interactome in HEK293T cells to establish and test the methodology and in naïve murine embryonic stem cells (mESC), in which the role of TDG in dynamic DNA methylation is well established. This approach identified several as-yet undescribed TDG-protein interactions associated with important genome functions and mechanisms beyond DNA repair and DNA demethylation. We report that TDG participates in a genome regulatory network through multiple interactions with epigenetic regulators, including also different RNA-binding proteins and even long-noncoding RNAs with a role in epigenetic regulation. Altogether, these findings provide novel insight into molecular pathways and mechanisms underlying the function of TDG-dependent active DNA demethylation in cellular plasticity and development.

## Materials and methods

### Cell culture

HEK293T cells (*WT or Tdg*^*−/−*^) were cultured in a controlled atmosphere (37°C, 5% CO_2_, 95% humidity) in DMEM (low glucose) supplemented with 10% FCS, 1 × L-glutamine, 1 × non-essential amino acids, 1 × HEPES and 1 × sodium pyruvate. 0.6 × 10^6^ of *Tdg*^−/−^ HEK293T cells were seeded in 6-well plates the day before transfection. For each sample (induced and not induced TDG-BirA*, NLS-BirA*, and H4-BirA*), an entire 6-well plate at 80% cell confluency was transfected using Lipofectamine 2000 (Life Technologies) following the manufacturer's protocol. Each transfection used 3 μg of plasmid DNA with BioID2 construct and 1 μg GFP-bearing plasmid DNA. 10 h post-transfection, cells transfected with the same plasmid DNA were pooled and re-plated into a 15-cm culture dish. After 24 h, cells were cultured in a medium supplemented with 50 µM biotin (VWR Life Science) for 24 h.

mESC were cultured under a controlled atmosphere (37°C, 5% CO_2_, 95% humidity) in serum-free 2i medium supplemented with 1’000 U/mL LIF, without antibiotics unless otherwise stated. *Tdg* depletion was done via Cre activation by tamoxifen (4-OHT), as described by Schwarz et al*.* [30]. Cells were treated with 3 µM 4-OHT (Sigma-Aldrich H7904) or mock-treated with DMSO in 2i medium for two hours before the medium was replaced.

### Molecular cloning

The MCS-BioID2-HA plasmid (Addgene #74224) was used for the cloning of the plasmids employed in BioID2 (pCMV-mTDGa-BioID2-HA, Addgene #232027; pCMV-NLS-BioID2-HA, Addgene #232025; pCMV-H4-BioID2-HA, Addgene #232026; pPgk-mTDGa-BioID2-HA, Addgene #232030; pPgk-NLS-BioID2-HA, Addgene #232031)*.* The mTDG (from plasmid Addgene #81050) and the replacement of CMV promoter with the murine *Pgk1* promoter were introduced using standard cloning methods based on PCR amplification with adaptor-oligonucleotides containing suitable restriction sites. For the generation of murine *Sra1* RNA, the pGEM-7zf(+)_mmSRA1 plasmid (Addgene #232032) was constructed by PCR amplification of reverse-transcribed mESC RNA using adaptor oligonucleotides. The product was cloned into the *Hin*dIII/*Eco*RI sites of pGEM-7zf(+) (Promega).

### Generation of stable BioID2-expressing mESC

0.3 × 10^6^ TDGiKO1.1 mESC [30] were transfected with linearized plasmids pPgk-mTDGa-BioID2-HA or pPgk-NLS-BioID2-HA, using Lipofectamine 2000 according to the manufacturer’s protocol*.* Three days post-transfection, cells were selected with 400 μg/mL G418 for 3 days and subsequently with 200 µg/mL G418 for additional 6 days. Colonies were then picked, transferred to a 96-well plate, and expanded in 2i/LIF medium. The expression of the BirA* constructs in transgenic colonies was confirmed by western blot analysis.

### HEK293T cell harvesting and enrichment of biotinylated proteins

#### BioID2 affinity purification

After rinsing with PBS, HEK293T cells from confluent cultures in 15-cm dishes were collected by scratching. Pelleted cells were washed with PBS and lysed with 540 μL lysis buffer I (50 mM Tris pH 7.4, 8 M urea, 1 mM DTT, 1 × Protease inhibitor Complete (PIC)) at room temperature (RT). The lysates were transferred in a 15 mL polystyrene tube, and 30 μL of 20% Triton X-100 was added before sonication for 10 min (30/60 s ON/OFF, Bioruptor Diagenode). The samples were diluted with 630 μL pre-chilled lysis buffer II (50 mM Tris pH 7.4, 1.4 M urea) and centrifuged in a 2 mL tube at 16′000xg at 4 °C for 10 min. The supernatant was transferred to a new tube and incubated with pre-equilibrated Streptavidin C1 Dynabeads (50 μL) at 4 °C with agitation for 2 h. While continuously agitating at RT, the beads were washed four times with wash buffer (50 mM Tris pH 7.4, 4 M urea, 1 × PIC) and three times with 50 mM ammonium bicarbonate (with 1 × PIC) before proceeding with on-bead trypsinization (see procedure below).

#### Co-Immunoprecipitation

Transfected cells were harvested, washed twice with PBS, and resuspended with 200 µL B100 Lysis buffer (10 mM Tris–HCl pH 7.5, 100 mM NaCl, 2 mM MgCl_2_, 0.5% Triton X-100) and 10 µL Benzonase. Cells were lysed on ice for 45 min followed by 15 min at 37 °C. Lysates were cleared by full-speed centrifugation for 5 min and diluted with 760 µL B100 buffer. 60 µL were saved as input fraction, while the remaining lysate was divided and incubated with either IgG1 (Biotechne) or HA Antibody (2–2.2.14, ThermoFisher Scientific), which were previously conjugated to Protein G Dynabeads (Invitrogen) with BS3 crosslinker and blocked with 1% gelatin in B100 buffer under rotation at 4 °C for 1 h. Beads were subsequently washed 4 × with B250 buffer (10 mM Tris–HCl pH 7.5, 250 mM NaCl, 2 mM MgCl_2_, 0.5% Triton X-100) and proteins eluted with 100 µL 1 × Laemmli buffer. 

### mESC nuclear extraction and enrichment for BioID2

2 × 10^7^ cells were treated with 4-OHT (pPgk-mTDGa-BioID2-HA*)* or DMSO (pPgk-NLS-BioID2-HA*,* parental mESC). After 48 h, each replicate (three or four replicates/cell line) was expanded in two plates and grown in 2i/LIF medium supplemented with 60 µM biotin for 48 h. For both approaches approx. 3 × 10^7^ cells were collected and washed twice with PBS.

- *Purification with denaturing conditions (approach A):* cells were gently resuspended in 200 μL Buffer A1 (10 mM HEPES pH 7.5, 10 mM KCl. 1.5 mM MgCl_2_, 340 mM sucrose, 10% glycerol, 1 mM DTT, 1 × PIC, 0.1% Triton X-100). After incubation on ice and centrifugation (4 °C, 1’300xg, 5 min), the cytoplasmic fraction (supernatant) was removed, and nuclei were washed once with Buffer A1 without Triton X-100 before snap-freezing. On the day of the purification, nuclei were lysed, sonicated for 20 min for and purified with 50 μL Streptavidin-T1 beads as described in BioID2 with HEK293T cells.
- *Purification with high salt conditions (approach B):* cells were resuspended with 500 μL Buffer A2 (10 mM HEPES pH 7.5, 10 mM KCl. 1.5 mM MgCl_2_, 1 × PIC, 1 mM DTT) and incubated on ice for 10 min. Swollen cells were then spun down (400xg, 5 min, 4 °C) and lysed with the help of a pestle (ca. 20 strokes) in 150 μL Buffer A2 supplemented with 0.15% NP-40. Nuclei were then collected by centrifugation (2'000xg, 10 min, 4 °C), washed once with Buffer A2 (without NP-40) and resuspended in 87 μL Buffer A2 (0.15% NP-40) and 12 μL of Benzonase (25 KU, Sigma, Cat. #71206–3). Genomic DNA digestion was carried out under rotation at 4 °C for 3 h. Subsequently, one volume (100 μL) of 2 × Buffer C2 (30 mM HEPES pH 7.5, 900 mM NaCl, 20% (v/v) glycerol, 0.4 mM EDTA, 2 mM MgCl_2_, 2 mM DTT) supplemented with 1 × PIC and 0.2% NP-40 was added, and nuclei were lysed by 20 pestle strokes and vortexing. After 1 h of incubation at 4 °C under rotation, cell debris was removed by centrifugation (20′000xg, 30 min, 4 °C). Supernatants were transferred into a new tube and diluted with 410 μL 1 × Buffer C2 and incubated with 50 μL Streptavidin T1-Dynabeads (Invitrogen, pre-blocked with 1% porcine gelatin at 4 °C for 1 h) at 4 °C overnight under rotation. Beads were subsequently washed for 10 min while rotating at RT with the following buffers; twice with wash buffer 1 (2% SDS in TE buffer with 1 mM DTT and 1 × PIC), once with wash buffer 2 (high-salt buffer: 50 mM HEPES pH 7.5, 1 mM EDTA, 1% Triton X-100, 0.1% Na-deoxycholate, 0.1% SDS, 500 mM NaCl, 1 mM DTT and 1 × PIC), once with wash buffer 3 (250 mM LiCl, 10 mM Tris pH 8.0, 0.5% NP-40, 0.5% Na-deoxycholate, 1 mM EDTA, 1 mM DTT, and 1 × PIC) and twice with TE buffer supplemented with 1 mM DTT and 1 × PIC at 4 °C for 10 min.

### On bead digestion, purification and LC-MS

Affinity-purified samples were subjected to on-bead tryptic digestion. Beads were first washed with a detergent-free wash solution (100 mM ammonium bicarbonate) and collected by centrifugation. Peptides were eluted by incubating in 1.6 M Urea, 100 mM ammonium bicarbonate, 5 µg/mL trypsin, pH 8 at 27 °C shaking at 1’200 rpm for 30 min followed by two washes in 1.6 M Urea, 100 mM ammonium bicarbonate, 1 mM TCEP, pH 8. After each wash step, the resin was spun down and supernatants were collected and pooled. TCEP and chloroacetamide were added to a final concentration of 10 mM and 15 mM, respectively, and samples were incubated at 37 °C shaking at 600 rpm for 1 h. After reduction and alkylation, 0.5 µg of trypsin was added, and samples were incubated at 37 °C shaking at 300 rpm for 12 h. The tryptic digest was acidified (pH < 3) using TFA and desalted using C18 reversed phase spin columns (Microspin, Harvard Apparatus) according to the manufacturer’s instructions. Peptides were dried under vacuum and stored at -20 °C. Dried peptides were dissolved in 0.1% aqueous formic acid solution at a concentration of 0.2 µg/µL before injection into the mass spectrometer.

For each sample, aliquots of 0.4 µg of total peptides were subjected to LC–MS analysis using a dual pressure LTQ-Orbitrap Elite mass spectrometer connected to an electrospray ion source (both Thermo Fisher Scientific) and a custom-made column heater set to 60 °C. Peptide separation was carried out using an EASY nLC-1000 system (Thermo Fisher Scientific) equipped with an RP-HPLC column (75 μm × 30 cm) packed in-house with C18 resin (ReproSil-Pur C18–AQ, 1.9 μm resin; Dr. Maisch GmbH, Germany) using a gradient from 95% solvent A (0.1% formic acid in water) and 5% solvent B (80% acetonitrile, 0.1% formic acid, in water) to 10% solvent B over 5 min, to 35% solvent B over 40 min to 50% solvent B over 15 min to 95% solvent B over 2 min and 95% solvent B over 18 min at a flow rate of 0.2 µL/min. The data acquisition mode was set to obtain one high-resolution MS scan in the FT part of the mass spectrometer at a resolution of 12′000 full width at half maximum (at 400 m/z, MS1) followed by MS/MS (MS2) scans in the linear ion trap of the 20 most intense MS signals. The charged state screening modus was enabled to exclude unassigned and singly charged ions, and the dynamic exclusion duration was set to 30 s. The collision energy was set to 35%, and one microscan was acquired for each spectrum.

### Chromatin fractionation and Western blot

Chromatin Fractionation was performed as described in [31]. For whole cell protein extraction, cells were harvested and washed with PBS. 1 × 10^6^ cells were mixed with 1 × Laemmli buffer (250 mM Tris–HCl pH 6.8, 40% Glycerol, 8% SDS, bromophenol blue) boiled at 95 °C for 10 min and sonicated for 10 min (30/60 s ON/OFF, Diagenode Bioruptor). Lysed cells were boiled at 95 °C for 2 min before loading the SDS-PAGE gel.

Resolved samples were wet-transferred (300 mA) to a 0.2 μm nitrocellulose membrane (GE Healthcare Life Science) at 4 °C for 2–3 h using transfer buffer (25 mM Tris-base, 193 mM glycine, 10% methanol). The membrane was blocked with 5% (w/v) BSA in TBS-T (10 mM Tris pH 7.5, 150 mM NaCl, 0.1% Tween-20) and incubated with the appropriate antibody diluted in blocking solution overnight at 4 °C or 1 h at RT. Membranes were washed three times with TBS-T and incubated with the species-specific horseradish peroxidase-conjugated secondary antibody (1:10’000, VWR). After several washing steps with TBS-T and substrate provision (WesternBright ECL, Advansta), immunoreactive bands were detected by the Syngene PXi Gel Imaging System.

### RNA immunoprecipitation

RNA immunoprecipitations were performed according to Sun et al*.* [32] with some modifications: 1.5 × 10^7^ mESC in 15-cm dishes were washed twice with PBS, crosslinked with 1% formaldehyde in 10 mL PBS at RT for 10 min, followed by quenching with 125 mM glycine. After three washes with ice-cold PBS, cells were scraped and harvested by centrifugation at 600xg at 4 °C for 8 min. The cell pellet was incubated for 10 min in 300 μL buffer A3 (5 mM PIPES pH 8.0, 85 mM KCl, 0.5% NP-40, 1 × PIC (Roche), SUPERase•In (50 U/mL, Ambion)). After one wash in buffer A3 without NP-40 nuclei were lysed in 750 μL buffer B3 (50 mM Tris–HCl pH 8.0, 1% SDS, 10 mM EDTA, 1 × PIC, SUPERase•In (50 U/mL)) on ice for 10 min. Lysates were sonicated (15/45 s ON/OFF) in a Bioruptor sonicator (Diagenode) at 4 °C for 15 min followed by centrifugation at 14′000xg at 4 °C for 10 min. Sonicates were diluted 1:10 in IP3 buffer (16.7 mM Tris–HCl pH 8.0, 0.01% SDS, 1% Triton X-100, 1.2 mM EDTA, 1 × PIC, SUPERase In (50 U/mL)) and precleared with pre-blocked Protein G beads (DYNAL/Invitrogen) at 4 °C for 1 h. An aliquot (2% of lysate) was preserved as input sample and stored at -80 °C until crosslink reversal. Immunoprecipitations were performed overnight at 4 °C containing either 2 μg affinity purified α-TDG antibody (0.16 μg/mL) or no antibody as control. Immune complexes were collected by incubation with pre-blocked Protein G beads at 4 °C for 2 h followed by 5 min wash steps at 4 °C with wash buffer I (20 mM Tris–HCl pH 8.0, 150 mM NaCl, 0.1% SDS, 1% Triton X-100, 2 mM EDTA), wash buffer II (20 mM Tris–HCl pH 8.0, 500 mM NaCl, 0.1% SDS, 1% Triton X-100, 2 mM EDTA) and wash buffer III (10 mM Tris–HCl pH 8.0, 0.25 M LiCl, 1% NP-40, 1% deoxycholate, 1 mM EDTA) and two washes with TE pH 8 buffer. Immune complexes were eluted twice with elution buffer (0.1 M NaHCO_3_, 1% SDS, SUPERase•In (50 U/mL)) at RT for 15 min under rotation. Crosslink reversal of immune-precipitates and input controls was performed by adding NaCl to a final concentration of 200 mM and incubating at 65 °C for 2 h, followed by the addition of Proteinase K (50 μg/mL) and incubation at 45 °C for 1 h. Samples were subjected to Trizol (Invitrogen) extraction according to the manufacturer's protocol. RNAs were treated with DNase I, reverse transcribed with RevertAid First Strand cDNA synthesis kit (ThermoFisher), and the qPCR was performed with SensiFAST SYBR No-ROX kit (Meridian Bioscience). Primers sequences are provided in Suppl. Table S1.

### Recombinant proteins and base-release assay

Recombinant TDG and APE1 were purified as described in Weber et al. [10]. Oligonucleotides were synthesized by Microsynth or supplied by Adam Robertson (5mC, 5hmC and 5caC-containing oligonucleotides). Oligonucleotide sequences are provided in Suppl. Table S1. Annealing of substrate strands was carried out in annealing buffer (10 mM Tris–HCl, pH 8.0, 50 mM NaCl) by heating to 95 °C (DNA) or 80 °C (RNA) for 10 min and ramping down to 4 °C at a rate of 0.02 °C/min. Double-stranded RNAs were prepared by annealing oligonucleotide 1 with oligonucleotide 2. DNA:RNA hybrids were generated using oligonucleotides 1 and 4, whereas RNA:DNA-labelled hybrids were formed using oligonucleotides 3 and 5 (Suppl. Table S1). The R-loop substrates were generated in two steps: first, oligonucleotides 3 and 5 were heated at 90 °C for 1 min, and the reaction was ramped down to 71 °C at a rate of 0.02 °C/min, then oligonucleotide 6 was added, and the reaction was ramped down to 4 °C.

For base-release assays using nuclear extracts, ca. 2 × 10^7^ nuclei were isolated as described in Steinacher et al. [31], and incubated in 12 μL buffer C4 (50 mM Tris–HCl pH 8.0, 0.1 mg/mL BSA, 0.2 mM EGTA, 3 mM EDTA, 420 mM NaCl, 1 × PIC, 1 mM DTT, 1 mM PMSF) under rotation for 1 h. After sonication and centrifugation at full speed at 4 °C for 15 min, extracted nuclear proteins were quantified with Bradford assay (Biorad). Extracted proteins (300 μg) or recombinant murine TDG (2.5 pmol) were incubated with heteroduplex substrates (1 µM) in a 50 μL nicking assay buffer (50 mM Tris–HCl pH 8.0, 1 mM DTT, 0.1 mg/mL BSA, 1 mM EDTA) overnight at 37 °C [10]. The reaction was stopped by Proteinase K (0.2 mg/mL) digest at 37 °C for 30 min, followed by the addition of 5 μL of NaOH (1 N) and incubation at 99 °C for 10 min. After EtOH precipitation overnight at -20 °C, the products were separated by denaturing 15% PAGE as described in Hardeland et al. [17].

For base-release assays using recombinant proteins, reactions were carried out with recombinant TDG (2.5 pmol) and substrate (2.5 pmol) in 10 μL BER buffer (50 mM HEPES pH 8.0, 70 mM KCl, 0.5 mg/mL BSA, 7 mM MgCl_2_, 1 mM DTT) with RNase inhibitor (NEB M0314) at 37 °C for 15 min, followed by 5 min of incubation with APE1 (2.5 pmol). The reaction was then stopped by the addition of 1 volume of 90% Formamide, 20 mM EDTA, 1 × TBE. Substrates were denatured at 95 °C for 4 min, immediately cooled down on ice and separated by denaturing (8 M urea) 20% PAGE. For the base-release reactions, which did not involve the detection of labeled RNA and the use of APE1, reactions were carried out in the nicking assay buffer and stopped by the addition of 1 M NaOH to a final concentration of 100 mM and heating at 99 °C for 10 min as described previously [17]. TDG time-course reactions were performed as described by Weber et al. [10]. Base-release in labeled substrate strands was detected by a Typhoon 9400 scanner (GE Healthcare) and analyzed quantitatively by ImageQuant TL software (v7.0, GE Healthcare).

### Electrophoretic mobility shift assay (EMSA)

Mouse *Sra1* and human *Neat1* RNA was in vitro transcribed with the AmpliScribe T7-Flash Transcription kit (Lucigen) as described in the manufacturer’s procedure from the template plasmids pCRII_TOPO-hNEAT1 (Addgene #61518) and pGem-7zf(+)_mmSRA1 (Addgene #232032) and purified with the RNeasy MinElute Cleanup kit (Qiagen). 180 ng RNA was incubated with indicated concentrations of recombinant TDG in BER buffer in the presence of RNase inhibitor at RT for 15 min. For competition assays, unlabeled DNA substrates (60 bp) containing a G·U mismatch (Subs60G·U) or a non-competitor homoduplex at indicated concentrations was added simultaneously or after 15 min. Subsequently, samples were run in a 1% agarose gel at 3 V/cm. For EMSAs with the 60 bp fluorescent-labeled substrates, 2 µM TDG was incubated at RT for 15 min with 2 µM substrate with or without 2 µM Subs60G·U unlabeled DNA substrate and separated by native 8% PAGE at 10 V/cm.

### Flow cytometry

Cells were harvested, washed twice with washing solution (PBS, 0.04% BSA) and filtered through a cell strainer (40 µm). 1 × 10^6^ cells were fixed with 100 µL 4% formaldehyde diluted in PBS at RT for 10 min and treated with 100 µL permeabilization solution (1% BSA, 0.1% Triton X-100, in PBS) at RT for 30 min. After centrifugation, cells were incubated with 100 µL HA antibody (Cell Signaling C29F4, 1:800) diluted in blocking solution (PBS, 1% BSA) at RT for 1 h. After three washing steps, cells were incubated with the secondary Alexa-488 anti-mouse (Invitrogen, 1:2′000) antibody diluted in blocking solution at RT for 30 min. Cells were washed three times, stained with DAPI (200 ng/mL in blocking solution) at RT for 10 min, washed again and analyzed with CytoFLEX (Beckman Coulter) cytometer.

### Immunofluorescence and proximity ligation assay

Cells were grown on a coverslip pretreated with 1% HCl, 70% ethanol. For immunofluorescence: after washing with PBS, cells were fixed with 4% formaldehyde at RT for 10 min. Cells were washed thrice by gradually changing the buffer to PBS with 0.02% Tween-20 (PBS-T). Permeabilization was performed with 0.5% Triton X-100 diluted in PBS at RT for 10 min. Subsequently, cells were blocked with 3% BSA in PBS-T for 1 h and incubated with antibodies against Nucleolin (ab134164, 1:500) or HA (Cell Signaling C29F4, 1:800) at RT for 1 h, followed by incubation with Streptavidin-FITC (ThermoFischer SA1001, 1:1′000) and secondary antibody (Alexa-647 or Alexa-596, Invitrogen 1:500) at RT in the dark for 1 h. After three washing steps with PBS-T, cells were incubated with DAPI diluted in PBS (200 ng/mL), washed with PBS and water, and mounted on slides with VectaShield (Vector Laboratories). For Proximity ligation assay: staining was performed as recommended by the manufacturer (Duolink®, Sigma-Aldrich). 1:500 diluted anti-HCFC1 (Bethyl Laboratory: A301-400A) and anti-GFP (Roche: 180303) antibodies were incubated at RT for 1 h. Stained cells were visualized with confocal SP5 (Leica) or with STELLARIS 8 (Leica) microscopes. Quantification of the nuclear or nucleolar signals was done with the ImageJ/Fiji macro QuaSI (Bauer CU, GitHub).

### Data analysis

For the BioID2 data generated in HEK293T cells, the acquired raw files were imported into the Progenesis QI software (v2.0, Nonlinear Dynamics Limited) for analysis. Peptide precursor ion intensities were extracted across all samples using default parameters. The resulting mgf-file was searched with MASCOT against a human database (downloaded from Uniprot on March 6, 2019) containing murine H4-BirA* and TDG-BirA* sequences. The search criteria were as follows: full tryptic specificity was required (cleavage after lysine or arginine residues, unless followed by proline); up to three missed cleavages were allowed; carbamidomethylation (C) was set as fixed modification, while oxidation (M) and acetylation (protein N-term) were applied as variable modifications; mass tolerance was set to 10 ppm (precursor) and 0.6 Da (fragments). The database search results were filtered using the ion score to set the false discovery rate (FDR) to 1% at both the peptide and protein levels, based on the number of reverse protein sequence hits in the dataset. Quantitative analysis from label-free quantification were processed using the SafeQuant R package v.2.3.2. [33], to obtain relative peptide abundance. This included global data normalization by equalizing the total peak/reporter areas across all LC–MS runs, data imputation using the knn algorithm, summation of peak areas per protein, and LC–MS/MS run, and the calculation of peptide abundance ratios. For quantification, only isoform-specific peptide ion signals were included. To meet assumptions of normality and homoscedasticity for linear regression models and t-tests, MS-intensity signals were transformed from the linear to the log-scale. Summarized peptide expression values were used for statistical testing of differentially abundant peptides between conditions. Here, empirical Bayes moderated t-tests were applied, as implemented in the R/Bioconductor limma package [34]. Protein-level p-values from condition comparisons were adjusted for multiple testing using the Benjamini–Hochberg method.

For BioID2 data generated in mESC, raw files were analyzed using MaxQuant (v1.6.17.0) [35] with default settings. In brief, the spectra were searched against a murine database (UniProt) containing NLS-BirA* and Tdg-BirA* sequences, along with commonly observed contaminants, using the Andromeda search engine [36]. Search parameter included full tryptic specificity (cleavage after lysine or arginine residues, unless followed by proline), allowance for up to three missed cleavages, carbamidomethylation (C) as fixed modification, and oxidation (M) and acetylation (protein N-term) were applied as variable modifications. Mass tolerance was set to 20 ppm (precursor) and 0.5 Da (fragments). Label-free quantification and match between runs option were enabled. Database search results were filtered to a false discovery rate (FDR) of 1% at both the peptide and protein levels. Statistic data analysis was based on the MaxQuant proteinGroups output file, processed with Perseus (v1.6.10.43).

All proteomics data has been deposited on MassIVE, part of the ProteomeXchange consortium, under the accession number PXD041712. For all the BioID2 datasets, principal component analysis (PCA) was performed with LFQ normalized intensity values. Singular value decomposition (SVD) was employed to examine covariance/correlations between conditions.

### Public ChIP-seq and ATAC-seq data analysis

ChIP-seq binding peak data of CBX5 (GSE97945) [37], DDX21 (GSE69140) [38], HCFC1 and ZC3H11a (GSE36030) [39, 40], NCL (GSM5514081) [41], NONO (PRJNA527295) [42], NPAC (GSE95671) [43], PSPC1 (GSM5529373) [44], RIF1 (GSE98256) [45], RUVBL1 and RUVBL2 (GSE160738) [46], SMARCA4 (GSE111264) [47], SMCHD1 (GSE65748) [48], TDG (GSE55657) [49], TET1 (GSE26833) [50], TRIM28 (GSE126238) [51], WIZ (GSE137285) [52], and R-loops [53] were converted to the mouse genome assembly mm10 by the liftOver function using R/Bioconductor rtracklayer package [54]. Peaks significantly enriched over input (by MACS2 with stringent thresholds of Q-value < 1e-5) were considered and an overlap peak list was generated when ChIP-seq binding peaks were available from several biological replicates. Colocalization analyses were performed by overlapping genomic coordinates of ChIP-seq peaks of candidate factors with TDG binding sites (± 1.5 kb from TDG peak). To avoid redundancy due to overlapping or closely spaced ChIP-seq peaks, all genomic intervals were first processed using the reduce() function from the GenomicRanges R package. This step merged overlapping or adjacent peaks within each dataset into non-redundant genomic regions prior to downstream comparison and visualization. To correlate chromatin accessibility, ATAC peaks in naïve mESC (*Tdg*^+/+^ and *Tdg*^−/−^) (GSE166962) were called using MACS2 with parameters -p 0.005 -q 0.005 -a 20.0 -j -m 30 -r -v -l 50 and densities over sites with R-loops (± 3 kb) were calculated using the R-package ChIPseeker.

## Results

### Establishing TDG-BirA*-tagging and BioID2 for TDG interaction mapping in HEK293T cells

**Fig. 1:**
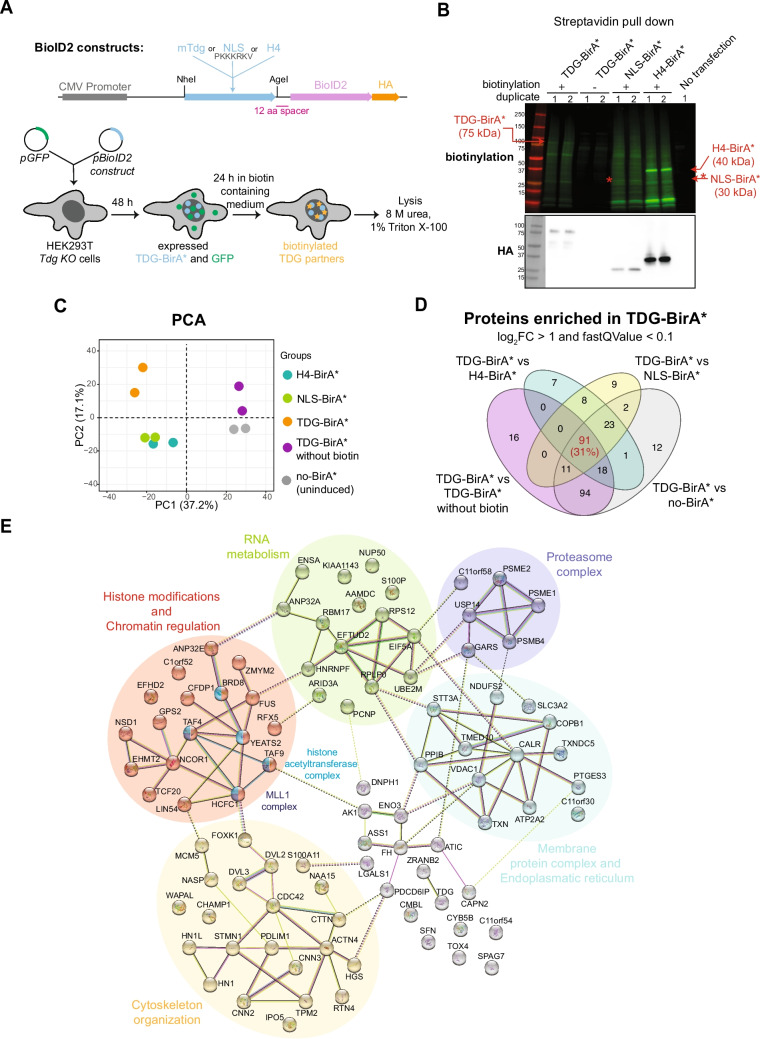
Identification of TDG interactors in HEK293T cells by BioID2-MS. **A** Schemes of BioID2 constructs and the experimental set-up. TDG-deficient HEK293T cells were transfected with the indicated BirA*-fusion constructs. 48 h after transfection, biotin (50 µM) was added to the cultures and cells were further incubated for 24 h before purification of biotinylated proteins from whole cell extracts. **B** Immunoblots detecting biotinylation (Streptavidin-800CW) (top) and levels of expression from transfected constructs (anti-HA antibody) in the eluates used for mass spectrometry. **C** Principal component analysis (PCA) of BioID2-MS data based on normalized intensity values of detected proteins. Coloring is set according to the experimental grouping. **D** Venn diagram of proteins enriched in TDG-BirA*-expressing cells compared to controls with a log_2_FC ≥ 1 and a fastQ-value < 0.1. **E** STRING network analysis of the 91 proteins identified in (D). The primary enriched protein clusters, determined using K-means clustering, are represented with distinct colors

Elucidating the mechanism coupling TET-TDG-mediated active DNA demethylation with chromatin landscaping requires more detailed insight into TDG’s environment of action. To provide this, we employed the BioID2 methodology and expressed *Tdg* fused to a promiscuous biotin ligase (BirA*) to facilitate biotinylation and subsequent identification of proteins in close proximity (within approx. 10 nm). Notably, BioID2 allows for the identification of both TDG-binding partners and transiently associating proteins, complementing previous approaches to identify TDG-protein interactions [13, 14, 20, 22]. We first used a HEK293T cell model to validate the suitability and functionality of TDG-BirA*-tagging for BioID2-facilitated identification of TDG-associated proteins. The TDG-BirA* fusion construct was further extended with an HA-tag to examine the relative expression level in cells. As controls, we used constructs expressing a nuclear localization signal-BirA* (NLS-BirA*) and histone H4-BirA* (H4-BirA*) fusion proteins, both HA-tagged, to identify proteins incidentally biotinylated in the nucleus or bound to chromatin independently of TDG (Fig. 1A). To control further for physiologically biotinylated proteins [55, 56] and non-specifically enriching proteins, we used cells not expressing any BirA* or cells expressing TDG-BirA* grown in biotin-free medium. Following transient transfection of HEK293T cells lacking endogenous TDG (*Tdg-KO*) and subsequent biotin labeling, total protein was extracted under denaturing conditions (8 M urea, 1% Triton X-100) (Fig. 1A). Biotinylated proteins were enriched using streptavidin affinity purification and analyzed by mass spectrometry-based relative quantitation. Transient expression of the BirA*-fusion proteins resulted in a substantial accumulation of biotinylated proteins when visualized with fluorescent-labeled streptavidin (Fig. 1B). Principal component analysis (PCA) of normalized intensity values of all detected proteins in mass spectrometry (Suppl. Table S2) segregated samples based on biotin supplementation in the first component (37.2% variation) and on TDG-BirA* expression in the second component (17% variation). H4-BirA* and NLS-BirA* controls clustered together (Fig. 1C). In total, 91 proteins were reproducibly enriched (log_2_FC > 1, q-value < 0.1) in cells expressing TDG-BirA* when compared to all controls (Fig. 1D, Suppl. Fig. S1A-D, Suppl. Table S2). Amongst them were transcription factors (ZMYM2, FOXK1, ARID3A), proteins involved in RNA metabolism, mRNA splicing/processing (FUS, RBM17, HNRNPF, EFTUD2), metabolic enzymes (FH, ASS1), components of the proteasome complex (PSME1, PSME2, PSMB4), and several proteins involved in histone modification and chromatin regulation (TAF4, TAF9, HCFC1, YEATS2, BRD8, EHMT2 and NSD1) (Fig. 1E). However, known TDG interactors, such as DNMT3A/B or TET proteins, were not represented in the dataset. Taken together, BirA*-tagging of TDG in HEK293T cells specifically identified proteins connecting TDG with RNA and chromatin biology.

### Identification of TDG-associated proteins in murine embryonic stem cells (mESC)

**Fig. 2:**
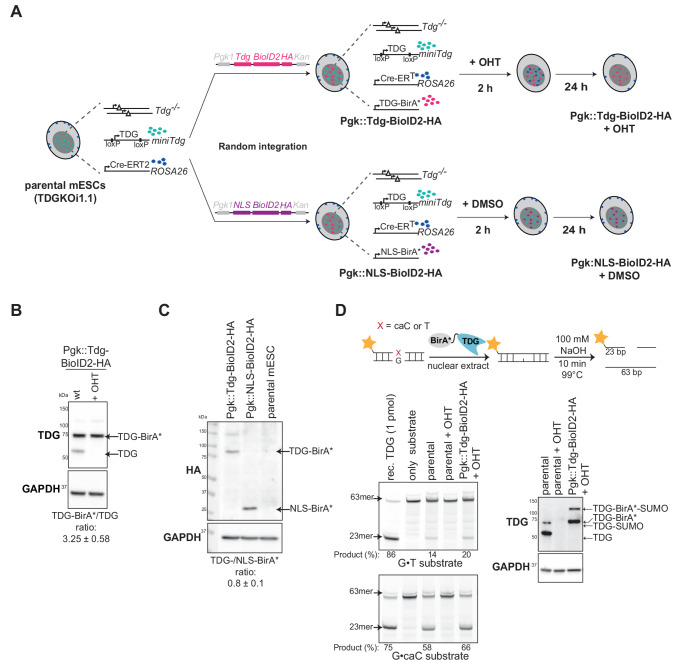
TDG-BioID2 model in mESC. **A** Generation of mESC lines. Plasmids bearing *Pgk1::Tdg-BioID2-HA* or *Pgk1::NLS-BioID2-HA* constructs were randomly integrated into the genome of TDGKOi1.1 mESC, containing a TDG mini-gene with loxP sites and a tamoxifen (OHT)-inducible Cre-ERT2 at the *ROSA26* locus [30]. **B**, **C** Immunoblots of whole cell lysates from mESC clones expressing HA-tagged TDG-BirA* (*Pgk::Tdg-BioID2-HA*) or NLS-BirA* (*Pgk::NLS-BioID2-HA*). Quantitation was done with ImageJ software from four replicates. **D** Base-release assays with G•T or G•caC containing substrates and nuclear protein extracts from TDG-BirA* expressing cells with and without depletion of the Tdg minigene (OHT treatment). Products were separated by denaturing DNA gel electrophoresis and visualized by fluorescent scanning (left panels). The status of TDG proteins was assessed by immunoblotting (right panel)

Next, we investigated the TDG interactome in naïve pluripotent mESC, where active DNA demethylation is ongoing [30, 57]. To assess protein proximity under physiological conditions and minimize artifacts caused by imbalanced protein concentrations, TDG-BirA* was stably expressed at near-endogenous levels in genetically TDG-depleted cells. This was achieved by ectopically integrating the murine Tdg-BioID2 expression construct under the control of the *Pgk1* promoter into the mESC genome carrying a single Cre-inducible *Tdg* disruption allele (*miniTdg*) [30] (Fig. 2A). This resulted in TDG-BirA* levels approximately three-fold (3.25 ± 0.58) higher than observed levels of endogenous TDG (Fig. 2B). As controls, mESC expressing the NLS-BirA* fusion protein from the *Pgk1* promoter or those not expressing any BirA* were used (Fig. 2C). To validate the activity and functionality of the TDG-BirA* fusion protein, we performed DNA base-release assays with nuclear extracts from mESC [58]. Synthetic DNA duplexes carrying either a defined caC•G base pair or a G•T mismatch were processed by TDG-BirA* as efficiently as by endogenous TDG (Fig. 2D). In addition, TDG-BirA* was post-translationally SUMOylated (Fig. 2B-D) and degraded in S-phase, similar to native TDG (Suppl. Fig. S2A, B). Altogether, these results showed that TDG-BirA*-HA behaves similarly to endogenous TDG and is suitable for generating a near-native TDG interactome.

**Fig. 3:**
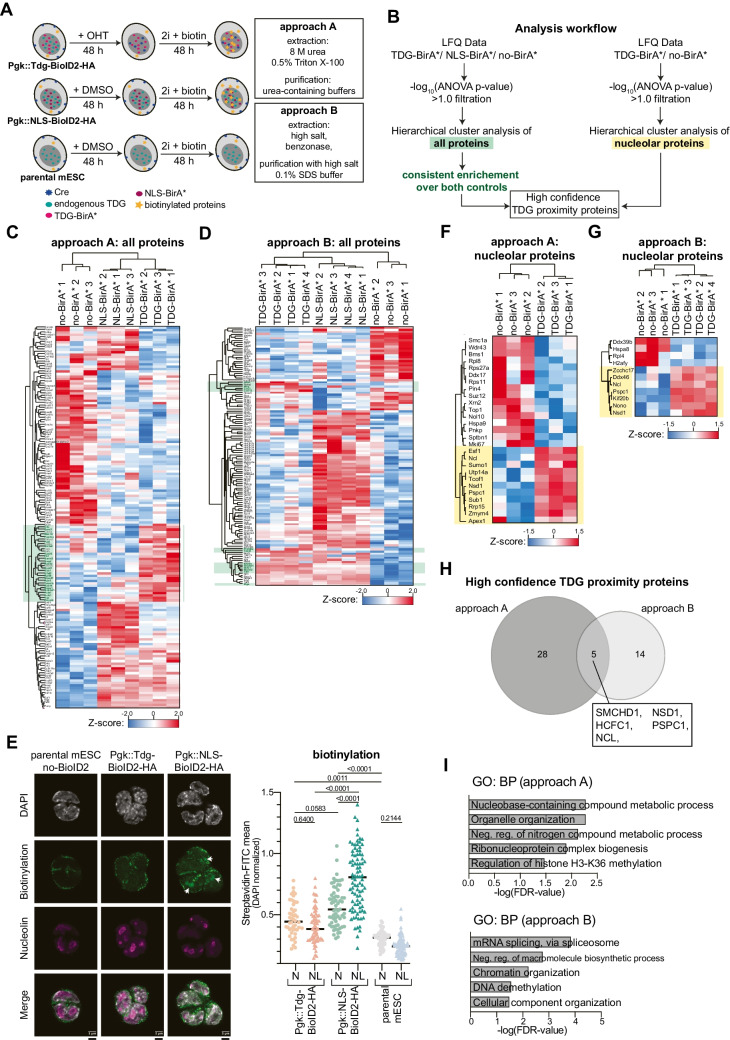
TDG-BioID2 screen in mESC. **A** Scheme of the experimental setup. Biotinylated proteins were purified by two methods (approach A and B) from nuclear fractions of transgenic mESC after 48 h of culturing in the presence of 60 µM biotin. **B** Work-flow of TDG-BioID2 proteomics data analysis illustrating the computational pipeline used to identify high-confidence TDG proximity proteins. BioID2-identified proteins were filtered by p-value (-log_10_(ANOVA p-value) > 1.0) and subjected to hierarchical cluster analysis. As high-confidence TDG proximity proteins were considered hits with consistent enrichment over both NLS-BirA* and no-BirA* controls. Nucleolar proteins were analyzed by comparing TDG-BirA* with no-BirA* control. **C**, **D** Hierarchical cluster analysis of all identified proteins in approach A/B. Proteins enriched in TDG-BirA* over both NLS-BirA* and no-BirA* controls are highlighted in green. **E** Confocal microscopy of mESC colonies after staining for biotinylated proteins by streptavidin-FITC, for the nucleolus by α-Nucleolin and DAPI counterstain of the nucleus. Scale bar = 3 μm. On the right, quantification (DAPI normalized) of the biotinylation signal in the different compartments. **F**, **G** Hierarchical cluster analysis of detected nucleolar proteins in approach A/B. Proteins enriched in TDG-BirA* are highlighted in yellow. **H** Venn diagram comparing high confidence proteins found in hierarchical cluster analysis in approach A and B. (**I**) Gene ontology analysis (Biological Processes) of significant proteins enriched in TDG-BirA*

Next, we used the mESC TDG-BirA* model to perform mass spectrometry of nuclear biotin-labeled proteins under defined conditions and in biological triplicates (Fig. 3A). Since TDG is degraded during the S-phase (Suppl. Fig. S2A and B) [59] and the mESC cell cycle ranges from 20 to 40 h, biotin (50 mM) was supplemented for 48 h to provide sufficient time for interactions with TDG (Fig. 3A). Notably, the lower expression of TDG-BirA* in mESC resulted in decreased overall biotinylation levels (Suppl. Fig. S3A) with a less pronounced difference in total biotinylation between TDG-BirA* and no-BirA* when compared to the over-expression system in HEK293T cells (Fig. 1B).

Protein extraction of the nuclear fraction and affinity purification were done under two different conditions to vary the stringency of extraction; (a) lysis and affinity purification using denaturing urea-containing buffers, as applied in the screen with the HEK293T cells, (approach A) [29], or (b) high-salt buffers for protein extraction, combined with high-salt buffer containing SDS for the washing steps during affinity purification (approach B) [60]. Hierarchical cluster analysis was conducted on reproducibly MS-detected proteins (with a -log_10_(ANOVA p-value) > 1.0) to identify proteins specifically enriched with TDG-BirA* over both controls (Fig. 3B). Approach A, involving stringent extraction conditions, identified 25 proteins (Fig. 3C), while approach B, involving stringent washing conditions, identified 13 proteins. The proteins identified by approach B showed a stronger enrichment, as exemplified by TET1 (Fig. 3D, Suppl. Fig. S3B-E, Suppl. Table S3).

Both, TDG-BirA* and NLS-BirA* showed a homogenous nuclear distribution in mESC (Suppl. Fig. S2B). Yet, NLS-BirA* preferentially biotinylated proteins within the nucleolus (Fig. 3E, Suppl. Fig. S3F). To mitigate a bias from the NLS-BirA* control, especially regarding nucleolar proteins, a separate analysis was conducted by comparing TDG-BirA* directly with cells lacking any BirA* expression (no-BirA*) (Fig. 3B). This targeted strategy identified 10 and 7 nucleolar proteins strongly biotinylated by TDG-BirA* in approaches A and B, respectively (Fig. 3F and G). Amongst them, SUMO1, previously identified as a Y2H partner of TDG [17], and AP-endonuclease 1 (APE1, APEX1), both of which known to cooperate with TDG in BER, were found, corroborating the validity and robustness of the experimental and analytical approach (Fig. 3F, Suppl. Fig. S3B, D).

**Fig. 4:**
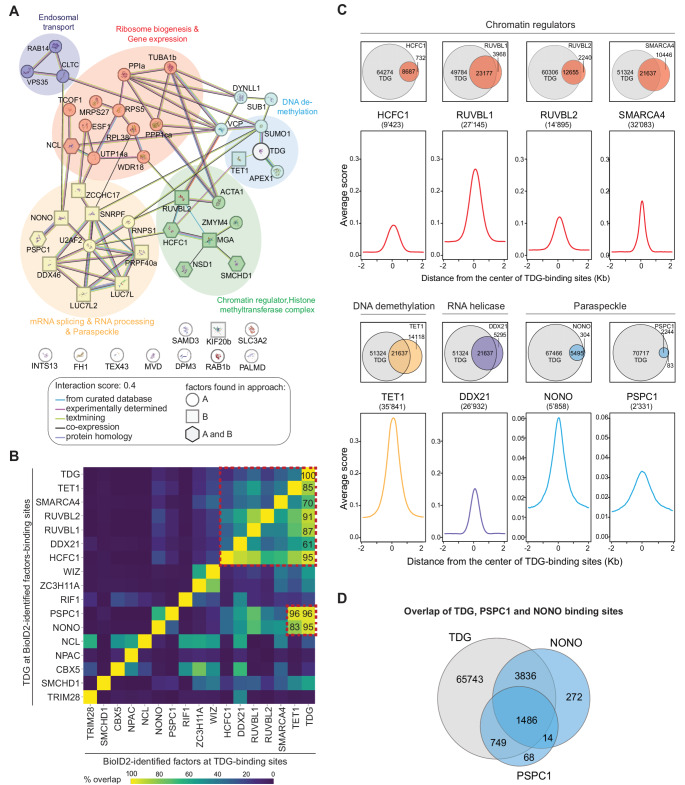
TDG protein proximity landscape in mESC. **A** STRING network analysis of the selected TDG proximity partners in mESC. The primary enriched protein clusters, determined using K-means clustering, are represented with distinct colors. **B** ChIP-sequencing overlaps between TDG and TDG-BirA* biotinylated proteins. Analysis includes only proteins with available ChIP-Seq data from mESC. The heatmap shows the percentages overlap of 17 distinct sets of candidate proteins’ chromatin associations (for data sets used, see Material and Methods), the scale corresponding to the percentage of overlap between chromatin associations of two proteins. Factors with high correlation of chromatin co-occupancy with TDG are framed in red. The numbers represent the percentage of overlap between TDG-binding sites and the chromatin-binding sites of the indicated proteins. **C** Density blot of the location of ChIP-seq peaks (lower panel) of selected proteins (TET1, HCFC1, RUVBL1, RUVBL2, SMARCA4, NONO, and PSPC1) relative to TDG-binding sites (73’006) identified in mESC (GSE55657). Histograms show the replicate average enrichment score within the region flanking (± 2 kb) the centre of TDG peaks. ChIP peaks that were significantly enriched over input (Q < 1e-5) were included and the number of peaks of each data set is indicated. Venn diagrams (upper panel) show the number of unique genomic regions bound by TDG and the respective factors. The number of regions in each dataset was reduced to non-overlapping intervals, which may result in fewer peaks than reported per ChIP-seq dataset. **D** Overlap of ChIP-seq peaks between TDG, NONO and PSPC1, indicating the proportion of co-occupancy

The different protein extraction and purification methods yielded distinct groups of TDG-BirA* proximity hits, demonstrating that cell lysis and purification conditions affect the spectrum of identifiable protein–protein interactions (Fig. 3H, Suppl. Fig. S4A-B). A total of 47 high-confidence TDG proximity proteins were identified using either approach A or B, including the 5 common hits SMCHD1, HCFC1, NSD1, PSPC1 and NCL (Fig. 3H). Gene ontology and protein network analyses using STRING revealed functional associations of identified proteins with chromatin organization, gene expression, RNA processing, and ribosomal and paraspeckle functions (Figs. 3I and 4A).

To substantiate the newly identified TDG interactions with chromatin-associated and/or gene regulatory factors, we analyzed the mutual chromatin co-occupancies with TDG using publicly available ChIP-seq data for these hits as well as related proteins. This analysis revealed that TDG colocalizes on genomic regions with several identified factors, including the chromatin regulators TET1, SMARCA4, RUVBL2, RUVBL1, DDX21, and HCFC1 as well as the paraspeckle-related proteins PSPC1 and NONO (Fig. 4B). Consistently, we found the individual ChIP-seq signals for these factors to peak at sites of TDG-chromatin association [37–53] (Fig. 4C). This colocalization suggests a physical and functional proximity between DNA demethylation activity by TET-TDG and various cellular processes: specifically, chromatin regulation (HCFC1, SMARCA4, RUVBL1, RUVBL2), and RNA processing/nuclear organization via the RNA helicase DDX21 and the paraspeckle proteins PSPC1 and NONO.

Among these interactions, HCFC1 was the one hit that appeared in all BioID2 experiments and replicates performed (Figs. 1E and 4A). HCFC1 is part of the MLL/COMPASS histone methyltransferase complexes responsible for the methylation of lysine 4 in histone H3 (H3K4me), an active chromatin mark previously found to be deregulated in TDG-deficient mouse embryonic fibroblasts (MEF) [5, 61]. Using GFP-tagged murine TDG (mTDG), we confirmed the vicinity of TDG and HCFC1 by proximity ligation assay (Suppl. Fig. S5A) in mESC and the physical interaction by co-immunoprecipitation (Co-IP) in HEK293T cells (Suppl. Fig. S5B), demonstrating that the C-terminal domain of HCFC1 can enrich TDG under the conditions tested. Furthermore, chromatin fractionation experiments in mESC and HEK293T cells revealed that the lack of TDG resulted in a reduction of the nuclear soluble fraction of HCFC1 (Suppl. Fig. S5C-D). This finding suggests a functional link between TDG and HCFC1 in the HCFC1-mediated regulation of chromatin dynamics. While TDG colocalizes substantially with the chromatin remodelers RUVBL1 and RUVBL2 (Fig. 4C), attempts to validate their direct interaction by Co-IP produced inconclusive results, suggesting that the mode and dynamics of in vivo interactions with these factors may be difficult to recapitulate in Co-IP experiments. Nevertheless, RUVBL1 as well as HCFC1 were found among the top 30 enriched proteins evidence from a previously reported TET1-BioID2 screen, supporting the relevance of these factors in the TET-TDG-mediated chromatin processes [62] (Suppl. Fig. S4C).

Similarly, the co-occupancy of genomic regions by TDG, PSPC1 and NONO, pointed to an association of TDG with processes involving RNA-binding and RNA metabolism (Fig. 4B and C). NONO and PSPC1 are both paraspeckle core proteins, of which PSPC1 was also identified in a TET1-BioID2 screen, corroborating its association with active DNA demethylation [62] (Suppl. Fig. S4C)**.** We observed a significant overlap between TDG binding sites and a large portion of the common NONO and PSPC1 binding sites (Fig. 4D). This suggests a potential role of TDG in processes at the intersection of DNA demethylation and RNA-related nuclear functions.

Altogether, these findings provide a detailed protein proximity map for TDG, implicating a strong involvement of active DNA demethylation in chromatin landscaping, whereby HCFC1 emerges as a key link between TDG and the regulation of activity states of chromatin and PSPC1 and NONO hint toward a link between TDG and paraspeckle function.

### NONO and PSPC1 interactions link TDG with long non-coding RNAs

**Fig. 5:**
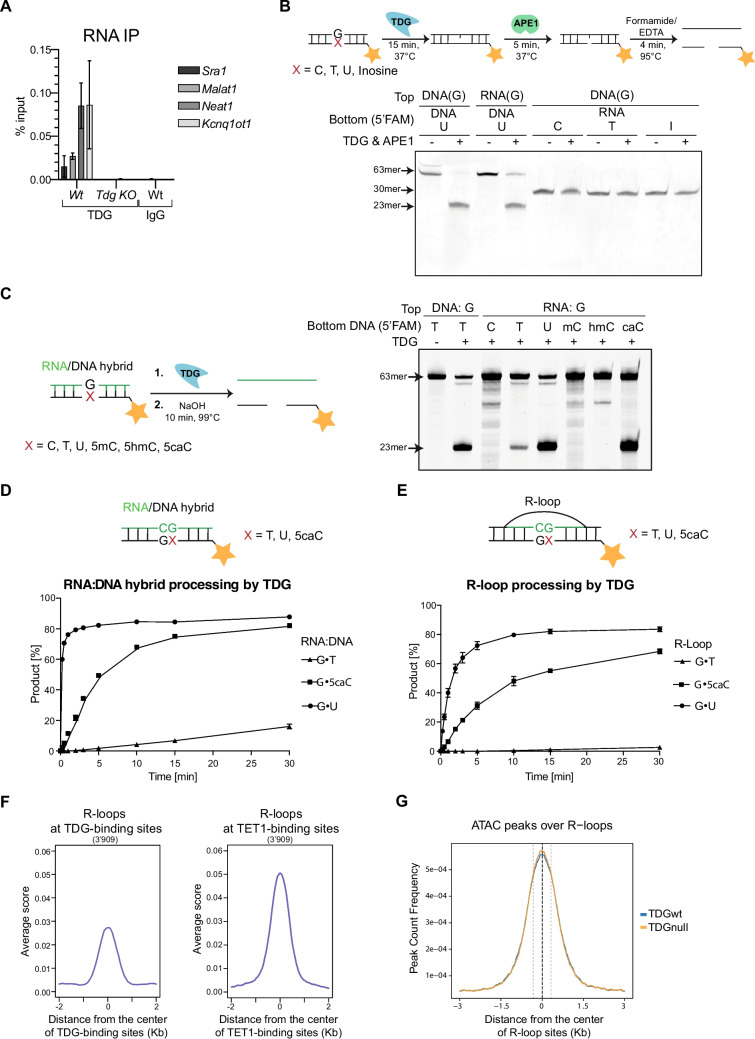
TDG binds RNA and processes DNA bases in R-loops. **A** TDG-RNA immunoprecipitation from mESC extracts. Cross-linked TDG-RNA complexes were purified using a TDG antibody. Shown are percentages of input with standard deviations (s.d.) (*n =* 3). **B** Base-release assay on 2.5 pmol RNA:DNA hybrid substrates, incubated with equimolar TDG for 15 min, followed by the addition of equimolar APE1 for 5 min. **C** Base-release assay with an RNA:DNA hybrid substrate; unlabeled 25 nt RNA strand was annealed to a complementary and labeled 60 nt DNA strand. Shown are the results obtained with substrates containing G•C, G•T, G•U, G•5mC, G•5hmC, or G•5caC base pairs. Reactions were carried out with equimolar TDG and stopped by adding NaOH. **D** Time-dependent generation of AP-sites by recombinant TDG (25 nM) was measured in reactions with equimolar concentration of 5’-FAM-labeled DNA:RNA hybrid substrates with mismatches/base-pairs indicated. Shown are mean percentages of product formation with s.d. (*n =* 3). **E** Base-release assay as described in D). The substrate used in this experiment is instead an R-loop substrate, as indicated. **F** Density blot of the location of ChIP-seq peaks (*n =* 3′909) of R-loops (GSE166426), which were significantly enriched over input (Q < 1e-5), at TDG-binding (GSE55657) or TET1-binding sites (GSE26833) identified in mESC. **G** Density plot depicting the overlap frequency of sites with significant enrichment in ATAC-seq with sites of R-loops. Grey dashed lines indicate the median size of R-loop enrichment, 646 bp)

Since the paraspeckle proteins NONO and PSPC1 emerged as significant hits and paraspeckle interactions are organized by the long non-coding RNA (lncRNA) *Neat1* [63]*,* we tested whether the observed proximity to TDG may be mediated by *Neat1* or other lncRNAs related to DNA repair, transcriptional control, or chromatin organization [63–66]. RNA immunoprecipitation showed a strong interaction of TDG with *Neat1* but also with other regulatory lncRNAs, such as *Sra1*, *Malat1,* and *Kcnq1ot1* (Fig. 5A), suggesting that lncRNA interaction might be a general feature of TDG. We then tested the ability of TDG to directly bind lncRNAs in electrophoretic mobility shift assays (EMSA) with in vitro transcribed *Neat1* and *Sra1* RNAs and observed robust binding to both lncRNAs (Suppl. Fig. S6A-D). Yet, TDG-lncRNA-binding occurred with an affinity lower than observed with double-stranded homoduplex DNA and G•U mismatched DNA substrates, as evident from competition experiments (Suppl. Fig. S6A, B, D)**.**

We next asked whether TDG is enzymatically active as an RNA glycosylase when bound to RNA. We tested the cleavage of a thymine that could potentially arise from 5mC deamination in RNA and of an inosine (I) that arises from A-to-I RNA editing of transcripts and, in the case of paraspeckles, regulates nuclear retention [67]. To test this, we used synthetic RNA substrates containing either a C, a T, or an I in a double- or single-stranded form. TDG bound all RNA-containing substrates efficiently and specifically, albeit with lower affinity compared to double-stranded DNA (dsDNA) substrates added as competitors to the EMSA (Suppl. Fig. S6E). Despite binding to the RNA substrates, TDG did not show RNA glycosylase activity on any of them, irrespective of their single- or double-stranded configuration (Suppl. Fig. S6F).

### TDG processes DNA:RNA hybrids in R-loops

Since lncRNAs and associated proteins can target and functionally interact with DNA [68, 69] through the formation of RNA:DNA hybrid or R-loop structures [70, 71], and since paraspeckle has been implicated in R-loop processing [72], we investigated whether TDG has the ability to process DNA or RNA in synthetic DNA:RNA hybrids. While there was no detectable RNA strand-directed base excision activity at G-paired T or I bases in the RNA strands, we observed significant TDG-dependent uracil excision activity in the DNA strands of U•G mismatched DNA:RNA hybrids (Fig. 5B). Further examination showed that TDG excises efficiently DNA caC (d5caC) and dU, and less efficiently dT when paired with an RNA G (rG) (Fig. 5C). We then analyzed the base-release kinetics to compare TDG processing ability on rG•d5caC, rG•dU, and rG•dT in RNA:DNA hybrid substrates. TDG removes d5caC and dU in RNA:DNA hybrids with initial rates comparable to dsDNA substrates; 11.40 and 94.5 [product %]/min for rG•d5caC and rG•dU, respectively, compared to 94.5 [product %]/min with dG•dT substrate [10]. By contrast, the rG•dT mismatch was processed at a much lower rate (0.53 [product %]/min) (Fig. 5D). We then assessed the ability of TDG to process these bases in the context of a synthetic R-loop substrate. TDG processed d5caC and dU paired with an rG within the R-loop with high efficiency (7.6 and 39.1 [product %]/min, respectively) but showed very poor activity on rG•dT (Fig. 5E). Altogether, these results show that TDG binds RNA and RNA:DNA hybrids and can process DNA bases in RNA:DNA hybrids, including R-loop substrates in vitro. Notably, these findings parallel available data on genome wide R-loop analyses in mESC [53], showing an enrichment of R-loops at TDG- and TET1-binding sites [50, 73] (Fig. 5F). The genome of naïve ES cells is generally highly accessible [74], while R-loops are associated with increased chromatin accessibility. Therefore, we investigated chromatin accessibility at R-loops in dependence of TDG. 153′853 significantly enriched ATAC-seq peaks were detected in wild-type mESC, while TDG depletion resulted in a global loss of more than 3′000 accessible regions. Interestingly enough, 20 R-loop regions gained accessibility in TDG-deficient cells (3′612 and 3′592 R-loop overlapping regions in TDGnull and TDGwt cells, respectively) [53], resulting in increased ATAC-seq signal density around the R-loop centers (Fig. 5G). Hence, these observations suggest a potential role for TET-TDG-dependent active DNA demethylation in regulation and processing of DNA:RNA hybrids and R-loops.

## Discussion

TDG is a DNA glycosylase with a central role in active DNA demethylation initiated by TET-catalyzed 5mC oxidation. While the molecular mechanism of active DNA demethylation is well understood [10], it does not explain its biological role in genome regulation and chromatin plasticity as implicated by the severe phenotype of a TDG defect on stem cell functionality and embryonal development [5, 75, 76]. We performed a comprehensive analysis of the TDG proximity network in mESC to provide novel insight into the chromatin and gene regulatory networks within which TDG operates. Besides corroborating the association of TDG with TET-dependent 5mC oxidation and the canonical BER pathway [10, 17], the newly uncovered protein interactions connect TDG with four genome functional aspects; chromatin organization and gene transcription, RNA-mediated nuclear processes and RNA-binding, ribosome biogenesis and function, and chromosomal organization. Notably, under the conditions used in our experiments, previously reported TDG interactors, identified by different methods, such as GADD45a [14], DNMT3A/B [13, 77], CREBBP/P300 [20], RAR/RXR [78] and ERα/β [25, 26], were not detected, indicating that these interactions may not be abundant and thus below the detection limit in naïve mESC, or that the TDG-BioID2 screen was not saturating for technical and/or biological reasons. This is in line with a recent TDG Co-IP study in murine stem cells by Aranda and colleagues, where these TDG interactors were also not detected [79]. Nevertheless, both screening approaches identified TDG proximity proteins falling into similar functional categories, including transcription factors, chromatin remodelers and RNA processing factors. The result of TDG-BioID2 screening in both mESC and HEK293T cells places TDG in contact with several important epigenetic regulators. This observation is consistent with previous findings that the absence of TDG imbalances activating and inactivating histone modifications in mouse embryonic fibroblasts (MEF) and, in particular, compromises targeted chromatin association of the H3K4-methyltransferase MLL1 or the histone acetyltransferase CREBP/P300 [5]. While there were no H3K4-specific methyltransferases (MLLs, KMTs) amongst TDG-BioID2 hits, we identified several other components of the MLL machinery, such as MGA, TAF9, and HCFC1, indicating that TDG interacts with the histone-modifying complex through these factors. We confirmed the TDG-HCFC1 interaction by immunoprecipitation and showed their colocalization on the chromatin (Suppl. Fig. S5, Fig. 4B-D). Notably, HCFC1 was also identified as an interaction partner of TET proteins in a TET1-BioID2 screen [62] (Suppl. Fig. S4C). Moreover, it was shown that HCFC1 undergoes GlcNAcylation and proteolytic processing by the O-GlcNac transferase OGT in a TET2/3-mediated manner to facilitate its maturation and association with the MLL1/COMPASS complex [80, 81]. It will be of great interest to further assess the mechanistic interplay between TDG-dependent dynamic DNA methylation and MLL function.

The screen further identified an interaction of TDG with the paraspeckle components NONO and PSPC1 (Fig. 4A and C). Notably, the two paraspeckle proteins were reported to be involved in the recruitment of TET proteins to chromatin [42, 44, 82]; while NONO was found to recruit TET1 to a subset of neuronal genes [42], PSPC1 was found to interact with TET1 and the lncRNA *Neat1* to modulate PRC2 binding at bivalent genes in mESC [44]. We show here that TDG is not only in close proximity to PSPC1 and NONO but it also binds *Neat1* directly, as well as other lncRNAs (Fig. 5A). Notably, we observed and reported previously that TDG depletion in MEFs leads to an apparent disbalance of H3K4 (MLL) versus H3K27 (PRC2) methylation at distinct gene loci (5). Hence, while our BioID2-screen implicates TDG in processes modulating MLL and PRC2 function, it will be important to reconcile TDG's activities in active DNA demethylation and lncRNA-binding with a possible mechanism balancing H3K4 and H3K27 methylation in cell differentiation.

Remarkably, we identified many additional RNA-binding proteins in direct proximity to TDG in both mESC and HEK293T cells, and we were able to enrich various lncRNAs by TDG immunoprecipitation in mESC (Figs. 1E, 4A and 5A). This indicates that TDG is an RNA-binding protein with a broad spectrum of substrates, consistent with recent findings of TDG binding to RNA molecules like *Hotair* and a *Tff1* enhancer transcript [83]. We therefore tested whether TDG would be active as a RNA glycosylase in various synthetic RNA and RNA:DNA hybrid substrates and were not able to detect any significant enzymatic activity (Suppl. Fig 5B, Fig. S6F). While these negative results are conclusive for the substrates tested (rT and rI base), they do not rule out an RNA-directed activity on other types of substrates. We were, however, able to demonstrate TDG activity in the DNA strand of RNA:DNA hybrids and R-loops (Fig. 5D and E). This is interesting in the light of observations that lncRNAs can act as scaffolds to target DNA demethylation factors to specific genomic sites. Research with HaCaT cells showed that TET2 and TDG associate with the lncRNA *Tetila*, which activates *Mmp9* transcription through DNA demethylation [84], whereas Arab et al*.* reported that R-loops can promote TET1-mediated 5mC oxidation through GADD45A [70]. The results presented here not only associate TDG with heteromolecular complexes shown to generate RNA:DNA hybrid structures and R-loops but also provide biochemical evidence for TDG being able to efficiently process TET-oxidized 5mC bases in a R-loop context. Recent data of Co-IP experiments indeed provided evidence for an in vivo association of TDG with R-loop structures [85]. This is consistent with the notion that lncRNAs can act as regulatory hubs for the DNA-demethylation machinery [86, 87], and it provides a mechanistic rationale for DNA strand-specific active DNA demethylation as occasionally observed [88]. It also may provide a rationale for how such “regulatory” R-loops can be resolved through BER-mediated DNA strand-incision and PARP activation [89, 90]. Yet, mechanistic and functional details remain to be elaborated in future investigations. Finally, it is worth noting that the association of mammalian TDG with RNA may be an evolutionary conserved function amongst vertebrates; it was previously observed that a chicken homologue of TDG, purified from chick embryos, needs RNA for 5mC-directed DNA demethylation, whereby the implicated function of the RNA was to target the enzyme to the site to be demethylated [91, 92]. These are interesting hypotheses and leads for further investigations towards dissecting the functional and mechanistic interplay between ncRNAs, RNA-binding proteins, active DNA demethylation, chromatin organisation and gene regulation in living cells.

Altogether our findings help to explain the pleiotropic phenotype of TDG-deficiency on gene expression, DNA methylation, chromatin modifications, cell differentiation, and mouse embryogenesis. Moreover, they provide valuable entry points towards unravelling the mechanisms coupling dynamic DNA demethylation with chromatin, RNA biology, and epigenetic programming of gene expression.

## Supplementary Information

Below is the link to the electronic supplementary material.

Supplementary file1 (PDF 3.51 MB)

Supplementary file2 (XLSX 13 KB)

Supplementary file3 (XLSX 879 KB)

Supplementary file4 (XLSX 347 KB)

## Data Availability

All proteomics data has been deposited on MassIVE, part of the ProteomeXchange consortium, under the accession number **PXD041712**. Data generated and analyzed during the current study are available from the corresponding authors upon reasonable request.
