# Supplementary material for: The TDG protein environment connects active DNA demethylation with chromatin and RNA biology
Source: Cell Mol Life Sci. 2025 Nov 25;83(1):15. doi: 10.1007/s00018-025-05943-y (PMC12748351; doi:10.1007/s00018-025-05943-y)
Supplement: Supplementary file 1 — Supplementary file1 (PDF 3.51 MB) [file 18_2025_5943_MOESM1_ESM.pdf]

The TDG protein environment connects active DNA demethylation with chromatin and RNA biology

Federica Richina[1], Faiza Noreen[1,2], Christina Bauer[1], Alain Weber[1], Christophe Kunz[1], Katarzyna Buczak[3], Simon D Schwarz[1], Fabian Wu[1], David Schürmann[1,*] and Primo Schär[1,*]

[1] Department of Biomedicine, University of Basel, Mattenstrasse 28, 4058 Basel

[2] Swiss Institute of Bioinformatics, 4031 Basel, Switzerland

[3] Proteomics Facility Biozentrum, University of Basel, Spitalstrasse 41, 4056 Basel

* Corresponding authors: david.schuermann@unibas.ch or primo.schaer@unibas.ch

Cellular and Molecular Life Sciences (2025)

https://doi.org/10.1007/s00018-025-05943-y

Supplementary Figures S1-S6

Supplementary Tables S1-S3

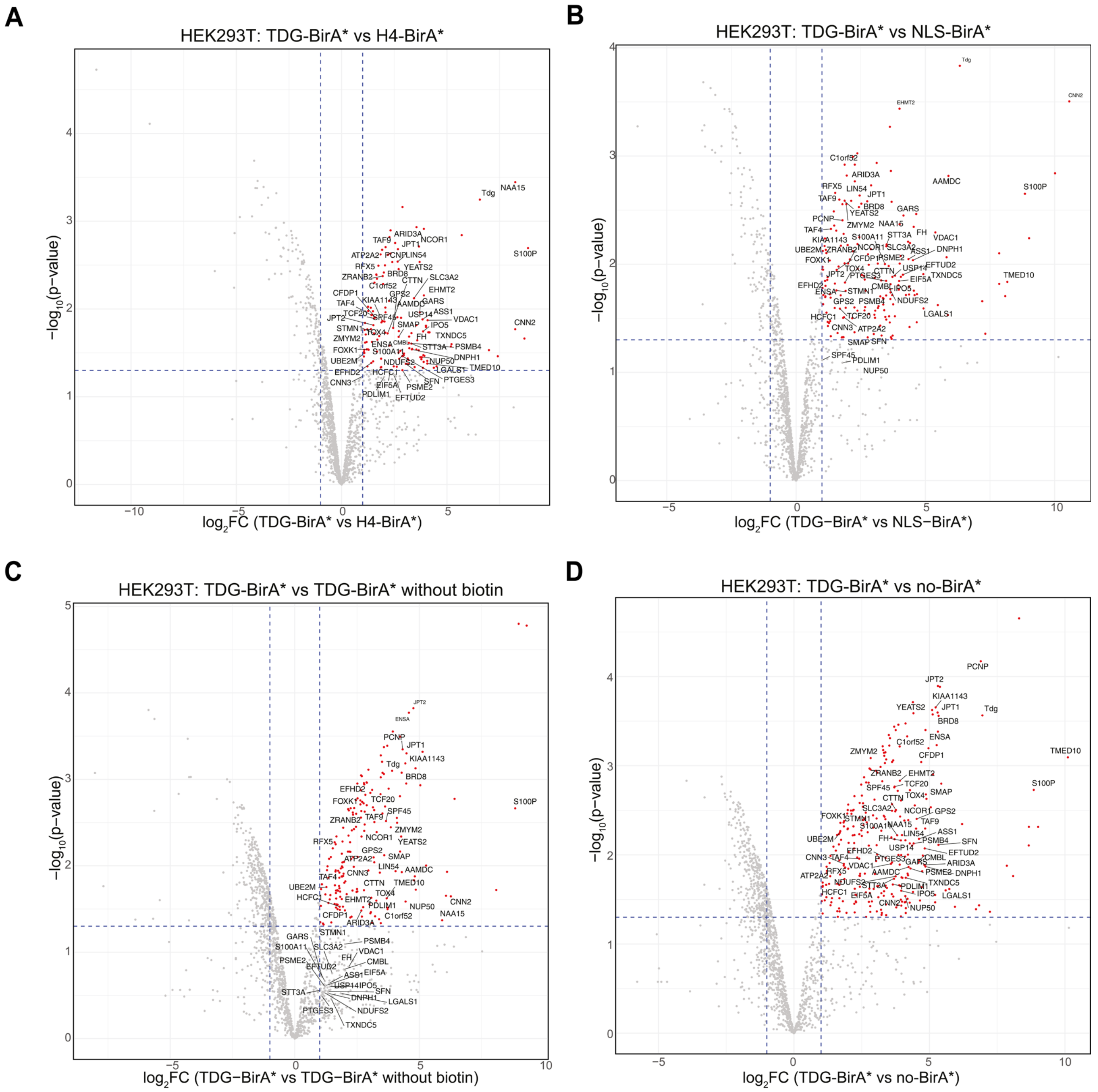

Suppl. Fig. S1 Identification of TDG interactors in HEK293T cells by BioID2-MS. (**A-D**) Volcano plots of proteins detected in HEK293T cells by BioID2 showing the $\log_2$FC of biotinylated proteins in TDG-BirA* compared to either H4-BirA*, NLS-BirA*, no-BirA*, TDG-BirA* without biotin (x-axis) and $-\log_{10}$ (p-value) (y-axis).

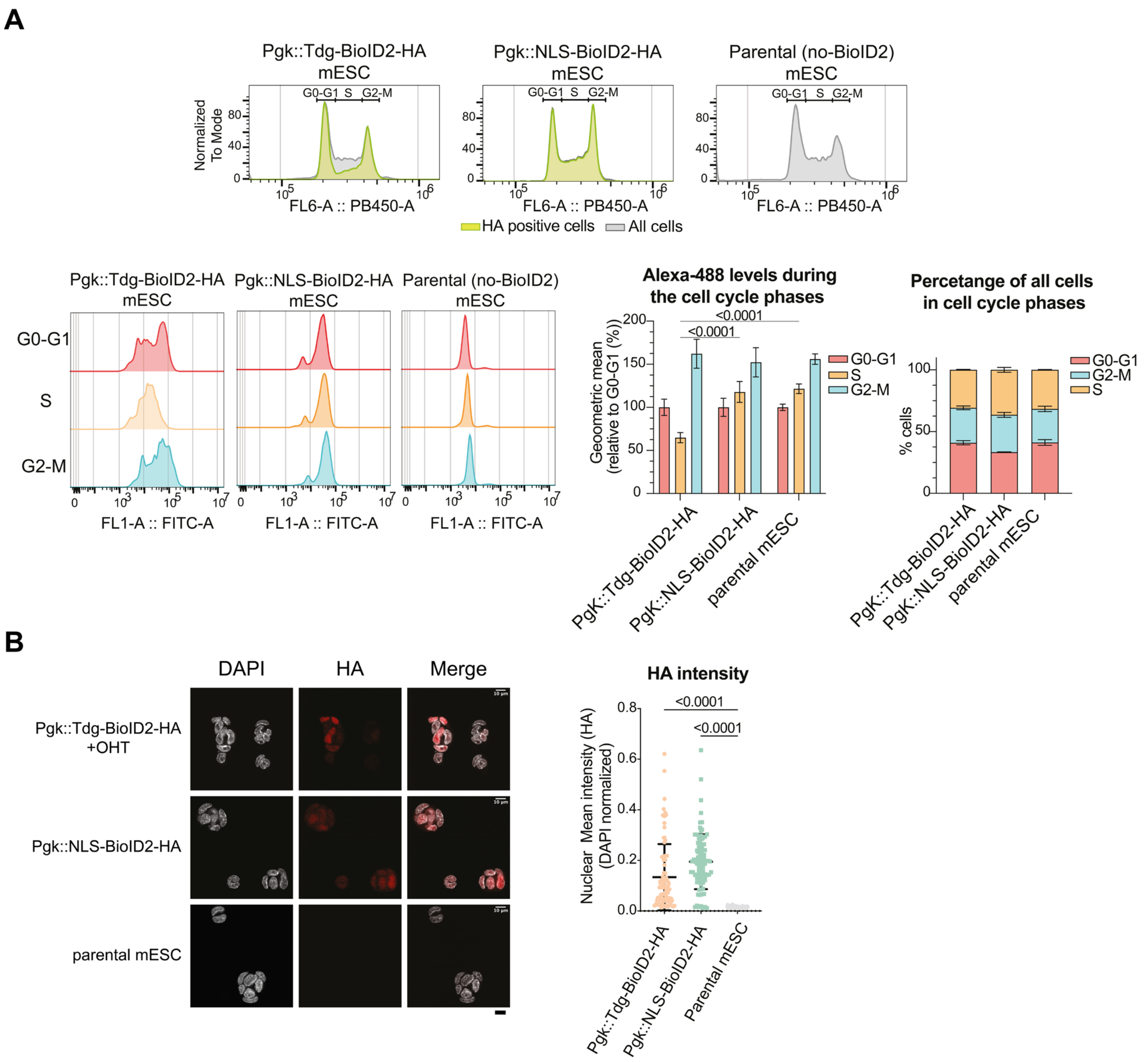

Suppl. Fig. S2 TDG-BirA* is degraded in S-Phase as the endogenous TDG. (**A**) Flow Cytometry of Pgk::Tdg-BioID2-HA or Pgk::NLS-BioID2-HA or TDGKO1.1 mESC lines, stained with HA (FITC) and DAPI. Cells were treated with 4-OHT or DMSO for 2 h and subsequently cultured for 48 h before immunodetection of HA-tagged proteins with anti-HA-Alexa488 antibody and incubation with DAPI to assess DNA contents and cell cycle stages. Analysis was carried out with a CytoFLEX (Beckman Coulter). (**B**) Confocal microscopy of mESC colonies after HA immunodetection and DAPI counter-staining of the nucleus. Scale bar = 10 μm.

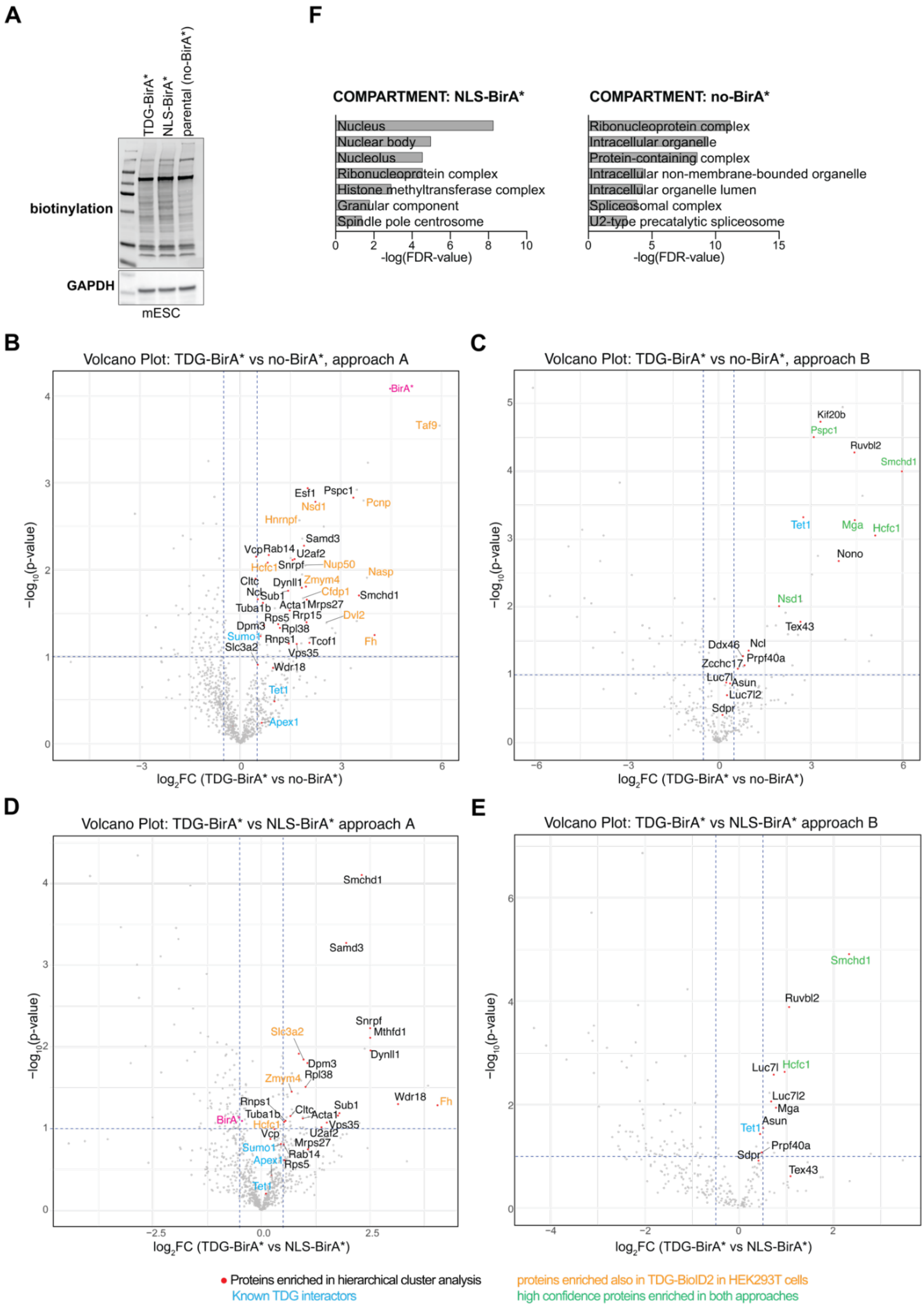

Suppl. Fig. S3 TDG-BioID2 screen in mESCs with the different controls. (**A**) Analysis of global biotinylation (Streptavidin-800CW) in whole cell extracts of cells expressing either TDG-BirA* or NLS-BirA* or no-BirA*. (**B-E**) Volcano plot of proteins detected in approach A or B, showing log_2FC of biotinylated proteins in TDG-BirA* expression mESC compared to either no-BirA* or NLS-BirA* (x-axis) and $-log_{10}$(p-value) (y-axis). Proteins found in hierarchical cluster analysis are marked as red dots. Known TDG interactors are indicated in blue, proteins identified in TDG-BioID2 in HEK2093T are orange, whereas proteins enriched in both approached are indicated in green. (**F**) COMPARTMENT enrichment analysis of proteins found in the two controls: Nuclear-free BirA* and no-BirA* (parental cell line).

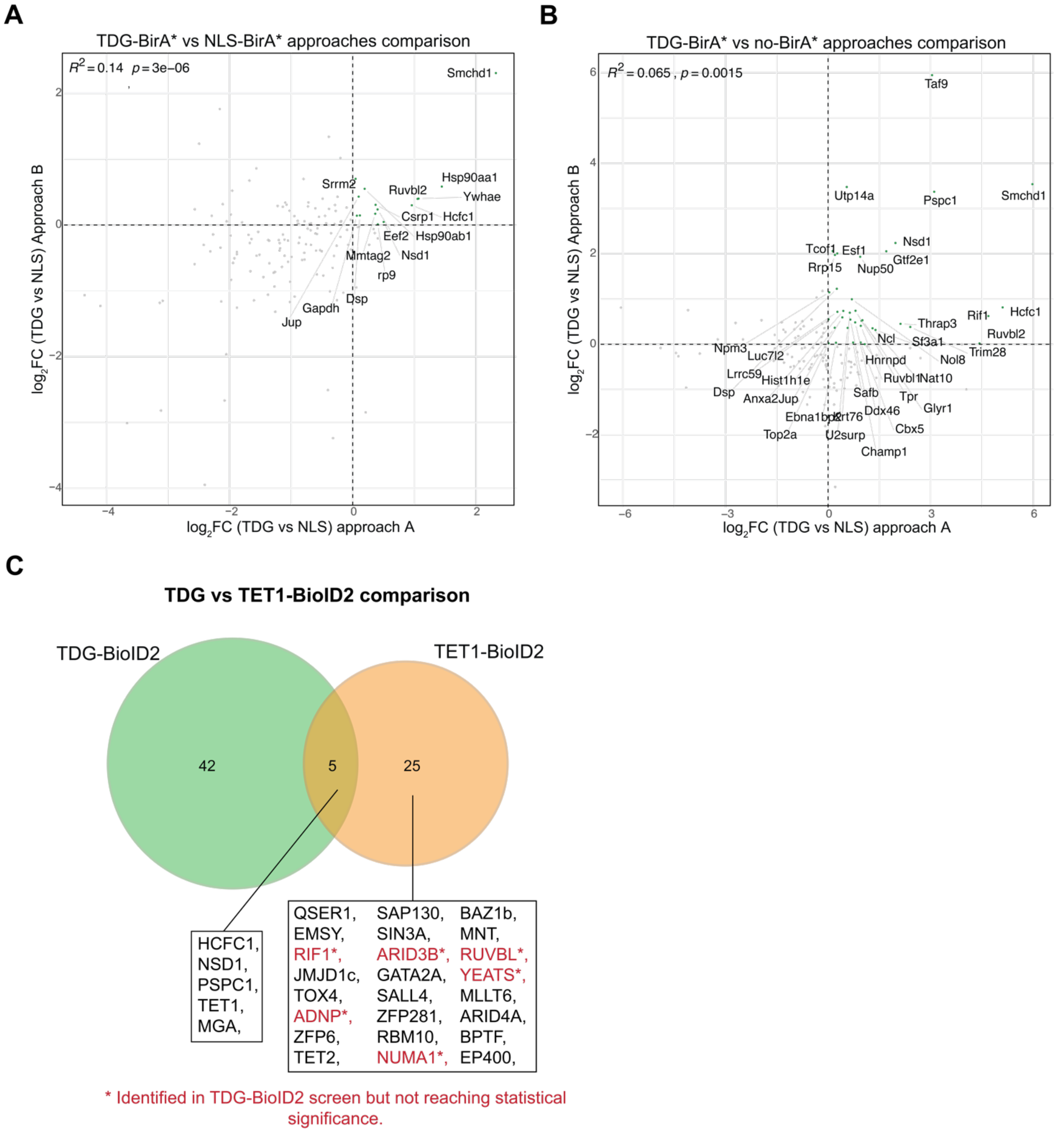

Suppl. Fig. S4 Comparison between approach A and B. (**A, B**) Scatter-plots comparing log_2FC (TDG-BirA* over either NLS-BirA* or no-BirA*) of proteins in approaches A and B. (**C**) TDG-BioID2 vs TET1-BioID2. Venn diagram of TDG-BioID2 (high confidence approach A+B) and TET1-BioID2 enriched proteins (FDR = 0.02) [1].

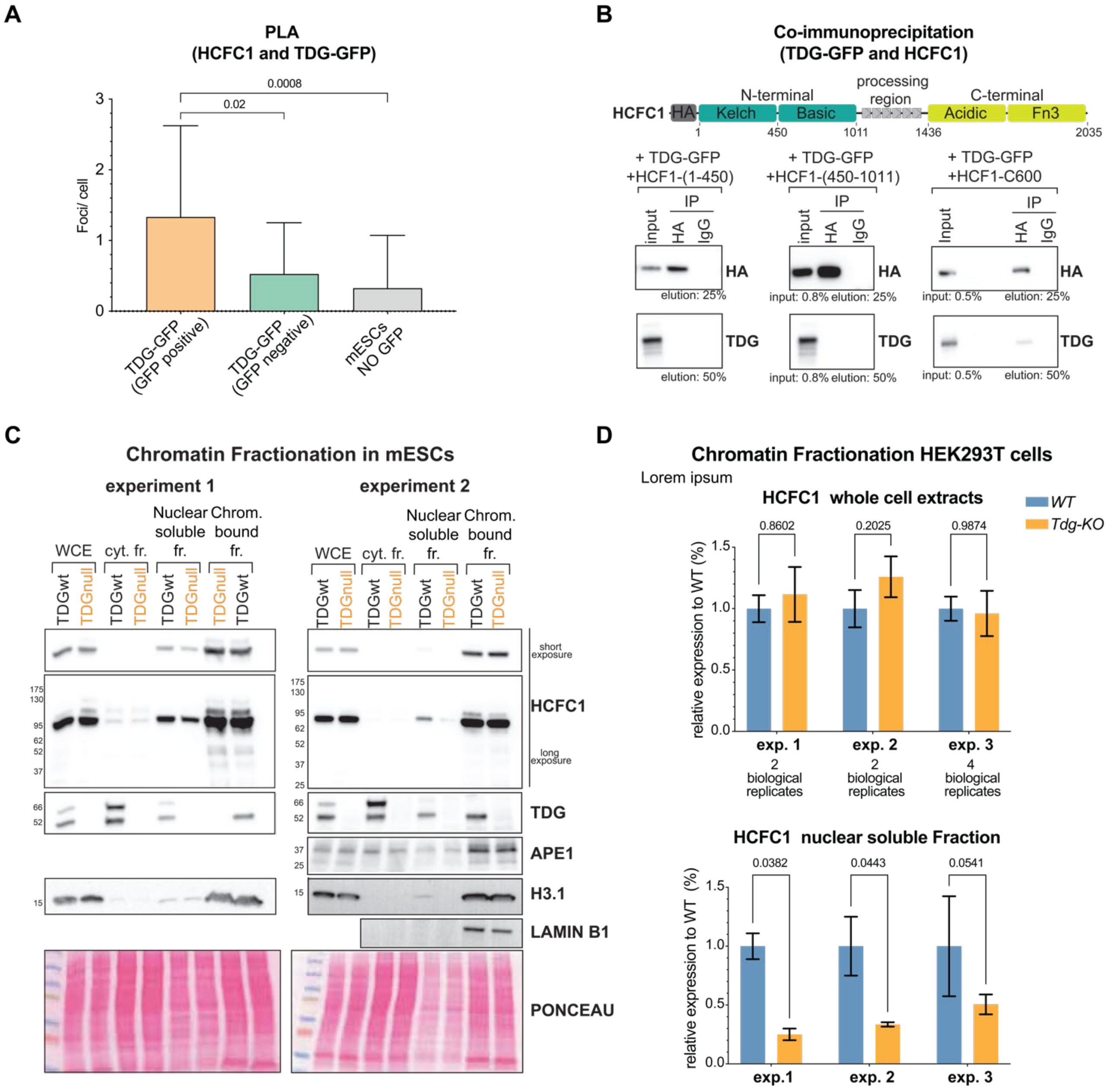

Suppl. Fig. S5 Physical and function interaction of HCFC1 and TDG. (**A**) Assessment of TDG-HCFC1 vicinity/interaction by Proximity Ligation Assay (PLA) in TDG-GFP expressing mouse embryonic stem cells (mESCs) [2]. The PLA was performed using anti-GFP and anti-HCFC1 antibodies. Mean foci number per cell ± standard deviation are shown. Cells lacking GFP expression were used as a negative control to assess background signal. (**B**) Validation of TDG-HCFC1 interaction by Co-IP with HA-tagged HCFC1 truncations (1-450, 450-1011, 1436-2035 (C600)) and TDG tagged with GFP, ectopically expressed in HEK293T cells. (**C**) Immunoblots of HCFC1 and controls of chromatin fractionation of TDG proficient (TDGwt) or deficient (TDGnull) mESCs, (n = 2). mESCs were treated either with DMSO (TDGwt) or 4-OHT (TDGnull) and were cultivated for 48 h in serum/LIF-containing medium before harvesting. (**D**) Quantitation of HCFC1 immunoblots of whole cell extracts (WCE) and nuclear soluble fractions in wild-type and *Tdg-KO* HEK293T cells. Data represent the mean ± standard deviation from three independent experiments, each with biological replicates.

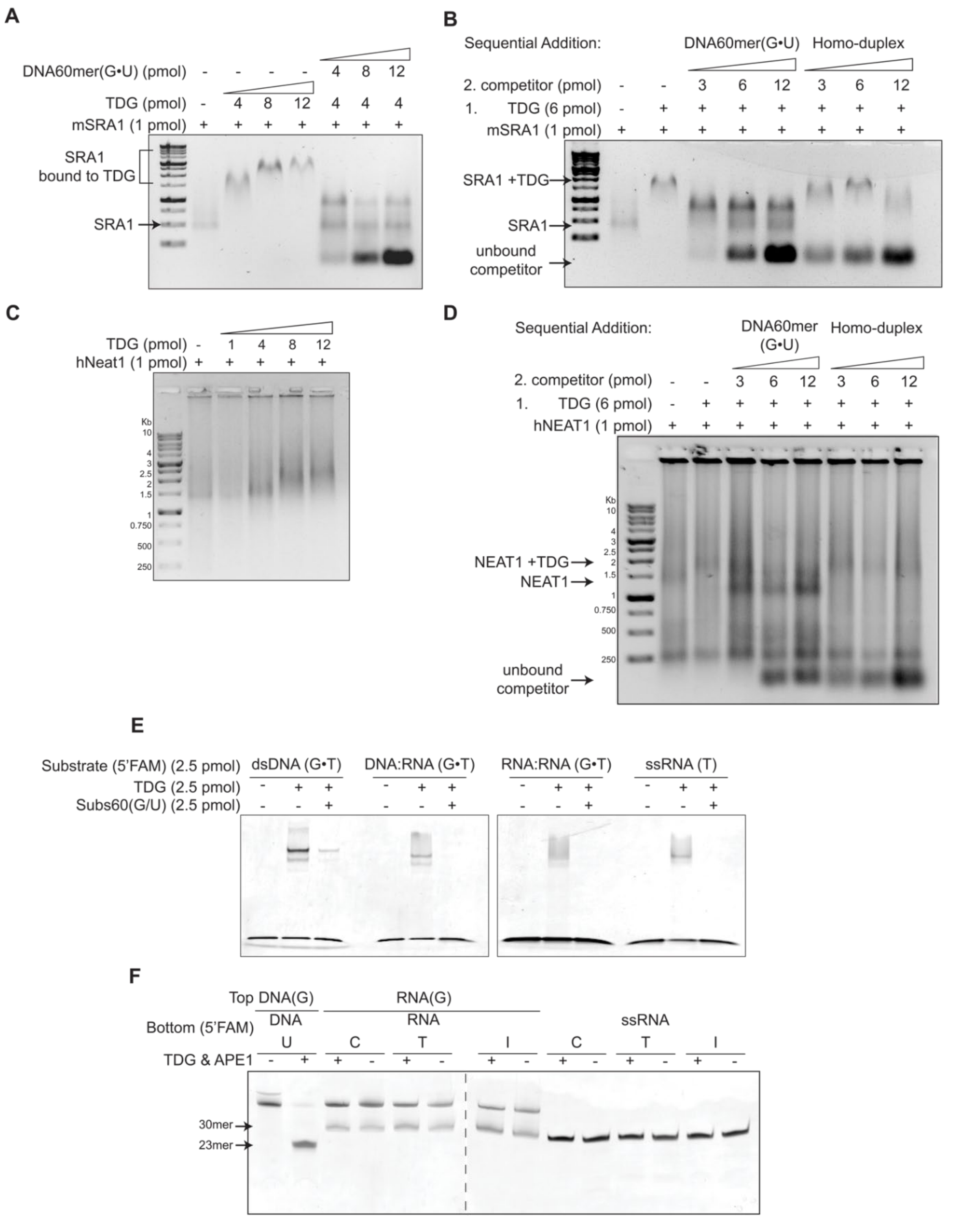

Suppl. Fig. S6 TDG binds but does not process RNA. (**A, C**) Nucleoprotein complex formation of recombinant TDG and RNA analyzed by EMSA. *mSra1* RNA and TDG were incubated together for 15 min at RT and as competitor either a 60mer G•U mismatched DNA substrate or a homoduplex DNA was added simultaneously (A) or 15 min after complex formation (B). (**B, D**) As in A and B), but executed with *in vitro* transcribed *hNeat1_1*. (**E**) EMSAs using FAM-labeled dsDNA, RNA:DNA, dsRNA or ssRNA substrates containing a thymine (T). 2.5 pmol TDG and 2.5 pmol substrates were incubated with or without 2.5 pmol of unlabeled 60 bp G•U dsDNA. Samples were separated on native 8% polyacrylamide gel. (**F**) Base-release assay with dsRNA or ssRNA substrates containing an inosine (I) or a thymine (T). Substrates were incubated with TDG (2.5 pmol) for 15 min, followed by APE1 (2.5 pmol) for 5 min and analyzed by denaturing 20% urea polyacrylamide gel electrophoresis.

Supplementary Table S1: Primers and oligo

This table consists of two sheets: one listing the primer sequences and the other detailing the oligonucleotide sequences used for the base-release and EMSA assays.

Supplementary Table S2: TDG-BioID2-MS data in HEK293T cells

This table contains a list of proteins identified through MS, their normalized intensity values, Log_2FC of TDG-BirA* against each control, and their associate $-log_{10}$(p-value) and q-value. In the last column ANOVA q-value was used as the cutoff for significant proteins.

Supplementary Table S3: TDG-BioID2-MS data in mESCs

This table comprises seven sheets, presenting data from the two TDG-BioID2 datasets generated using approaches A and B. It includes a comprehensive list of proteins identified through MS (both all proteins and nucleolar), along with normalized LFQ values, Log_2FC of TDG-BirA* against each control, and their corresponding $-log_{10}$(p-value) and q-value. The final sheet contains the high-confidence proteins selected for STRING network analysis.

Supplementary References

1. Karg E (2018) Investigation of the epigenetic protein landscape using proteomics-based strategies. Dissertation, LMU München: Faculty of Biology. Ludwig-Maximilians.Universität München
2. Steinacher R, Barekati Z, Botev P, et al (2018) SUMOylation coordinates BERosome assembly in active DNA demethylation during cell differentiation. EMBO J. https://doi.org/10.15252/embj.201899242
